# The Impact of Regulations, Safety Considerations and Physical Limitations on Research Progress at Maximum Biocontainment

**DOI:** 10.3390/v4123932

**Published:** 2012-12-19

**Authors:** Amy C. Shurtleff, Nicole Garza, Matthew Lackemeyer, Ricardo Carrion, Anthony Griffiths, Jean Patterson, Samuel S. Edwin, Sina Bavari

**Affiliations:** 1 Integrated Toxicology Division, United States Army Medical Research Institute of Infectious Diseases, 1425 Porter Street, Fort Detrick, MD 21702, USA; E-Mails: amy.c.shurtleff.ctr@us.army.mil (A.C.S.); nicole.garza@us.army.mil (N.G.); 2 Integrated Research Facility Frederick, Division of Clinical Research, National Institute of Allergy and Immunology, National Institute of Health, Frederick, MD 21702, USA; E-Mail: matthew.lackemeyer@nih.gov; 3 Department of Virology and Immunology, Texas Biomedical Research Institute, San Antonio, TX 78245, USA; E-Mails: carrion@txbiomed.org (R.C.); agriffiths@txbiomed.org (A.G.); jpatters@txbiomed.org (J.P.); 4 Select Agent Management Division, United States Army Medical Research Institute of Infectious Diseases, 1425 Porter Street, Fort Detrick, MD 21702, USA; E-Mail: samuel.edwin@us.army.mil

**Keywords:** biocontainment, biosafety level 4 (BSL-4), biological select agents and toxins (BSAT), positive pressure suit, biodefense, biosecurity, ebola virus, highly pathogenic viruses, limitations, collaboration

## Abstract

We describe herein, limitations on research at biosafety level 4 (BSL-4) containment laboratories, with regard to biosecurity regulations, safety considerations, research space limitations, and physical constraints in executing experimental procedures. These limitations can severely impact the number of collaborations and size of research projects investigating microbial pathogens of biodefense concern. Acquisition, use, storage, and transfer of biological select agents and toxins (BSAT) are highly regulated due to their potential to pose a severe threat to public health and safety. All federal, state, city, and local regulations must be followed to obtain and maintain registration for the institution to conduct research involving BSAT. These include initial screening and continuous monitoring of personnel, controlled access to containment laboratories, accurate and current BSAT inventory records. Safety considerations are paramount in BSL-4 containment laboratories while considering the types of research tools, workflow and time required for conducting both *in vivo* and *in vitro* experiments in limited space. Required use of a positive-pressure encapsulating suit imposes tremendous physical limitations on the researcher. Successful mitigation of these constraints requires additional time, effort, good communication, and creative solutions. Test and evaluation of novel vaccines and therapeutics conducted under good laboratory practice (GLP) conditions for FDA approval are prioritized and frequently share the same physical space with important ongoing basic research studies. The possibilities and limitations of biomedical research involving microbial pathogens of biodefense concern in BSL-4 containment laboratories are explored in this review.

## 1. Introduction

Laboratory personnel safety is of the utmost importance when working with any potentially infectious pathogenic organism. The United States Department of Health and Human Services (DHHS) has published guidelines recommending best practices for working safely with microbial pathogens by classifying organisms, designating biosafety levels (BSL) for use, elaborating on risks associated with handling various microbial agents, engineering controls to mitigate risks, and appropriate personal protection equipment (PPE) needed for laboratorians conducting scientific research in biocontainment laboratories [1]. Biosafety levels (BSLs) ascribe the proper microbiological practices, engineering controls, and safety practices required for a biocontainment laboratory, and the microbiological agents which can be handled safely within. Biosafety level 1 (BSL-1) confers the basic level of protection and is appropriate for microbial agents not known to cause disease in healthy humans. BSL-1 laboratories are often utilized for teaching and training of new laboratory personnel. Biosafety level 2 (BSL-2) laboratories contain moderate-risk agents that can cause human disease, albeit moderately, with variable disease course dependent on route of exposure. Most clinical, diagnostic, and teaching laboratories, where blood or blood products may be handled are BSL-2 laboratories. Agents handled at Biosafety level 3 (BSL-3) are known to cause potentially lethal infections and severe disease in humans. These agents pose a risk of disease through potential aerosol transmission and can be indigenous or exotic. The United States Department of Agriculture (USDA) has developed animal BSL-3 laboratories, classified as BSL-3-Ag, where research with high-risk pathogens which affect agricultural animals, crops or even humans can be executed. For these laboratories, containment is designed to protect the researcher and to prevent the release of agricultural pathogens into the surrounding environment to protect the local agriculture, and therefore the economy. Generally, agents handled at BSL-3 respond to medical countermeasures, lessening the disease severity. In contrast, pathogens handled at biosafety level 4 (BSL-4) pose a high risk of severe disease and have a higher potential for fatality. Unlike for pathogens handled at BSL-3, approved therapeutics and vaccines for prophylaxis do not exist. Due to the lack of medical countermeasures against these high‑risk agents and the potential for their weaponization; some of these pathogens are listed as National Institute of Allergy and Infectious Diseases (NIAID) Category A-C Priority Pathogens, and Centers for Disease Course and Prevention (CDC) Bioterrorism Agents in the United States [1,2]. Examples of such agents used within BSL-4 containment laboratories include filoviruses (ebola- and marburgviruses), arenaviruses (Lassa virus, Lujo virus, Chapare virus, Junín virus, Machupo virus, Guanarito virus, Sabiá virus), flaviviruses (tick-borne encephalitis virus, Omsk hemorrhagic fever virus, Kyasanur forest disease virus, Alkhurma hemorrhagic fever virus), bunyaviruses (Crimean-Congo hemorrhagic fever virus), henipaviruses (Hendra virus, Nipah virus), and variola virus. 

To advance our knowledge of these infectious agents, ongoing extensive laboratory experimentation is required. However, safety, security, and accountability measures must accompany this type of research due to the high risk levels associated with each agent’s respective disease course. The recognition of the threat of terrorism to the United States of America led to the USA PATRIOT act of 2001 to deter and punish perpetrators of terrorist acts in the United States and around the World. Regulations specifically on BSAT began with the United States Congress enacting the “Public Health Security and Bioterrorism Preparedness and Response Act” in 2002 to improve the ability of the United States to prevent, prepare for, and respond to bioterrorism and public health emergencies. Key DHHS regulation 42 Code of Federal Regulations (CFR) part 73, Select Agents and Toxins governs possession, use and transfer of select agents that have the potential to pose severe threats to public health and safety. The recombinant DNA Advisory Committee (RAC) through the Office of Biotechnology Activities (OBA) monitors manipulation of genetic material using recombinant DNA techniques. In addition, government agencies working with BSAT have developed and implemented their own additional, more stringent safety, security and agent accountability programs. The Department of Defense established a security directive ‘Safeguarding Biological Select Agents and Toxins’ (United States Department of Defense (DOD) directive 5210.88) for military laboratories with BSAT [3,4]. The Department of Army (DA) developed and implemented Army Regulation 50-1 (AR 50-1), a comprehensive Biological Surety program for all DA laboratories working with BSAT. The National Institute of Health (NIH) and the Department of Homeland Security (DHS) have similar programs with varying degrees of stringency for their laboratories working with BSAT. All institutions registered with CDC to work with BSAT implement and manage all aspects of federal, state, city, and local regulations through their own ‘Select Agent Program’ (SAP, [5]). These programs are similar and monitor four main criteria: biosafety, physical security, agent accountability, and personnel reliability [3]. Institutions registered with CDC to work with BSAT are inspected annually or as often as needed to ensure entities have policies and procedures to implement and abide by all current regulations. Inspecting agencies include CDC, United States Department of Agriculture (USDA), Department of Transportation (DOT) and the Department of the Army Inspector General (DAIG) for DA laboratories working with BSAT. The main purpose of all these logistical measures is to maintain safety, security and accountability of BSAT while conducting research within biocontainment. BSAT inventory management and tracking systems have been put into place to account for the infectious material present in the labs, in the form of stored vials, banked virus stocks, stored infectious samples, and working stocks of BSAT. These inventories are routinely inspected and cross checked for confirmation against the physical inventory during institutional inspections by regulatory inspecting agencies (e.g., annually). In addition, deemed export regulations apply to foreigners performing studies and collecting data in BSL-4 labs in the United States.

Currently, a handful of functional BSL-4 laboratories exist within the United States with a few newer laboratories undergoing rigorous testing and inspections to become certified and functional. Globally, there are many laboratories in countries such as Australia, Brazil, Germany, Gabon, Italy, Japan, Taiwan and Sweden where glove cabinets or full suit laboratories may be in service for BSL-4 research [6]. Table 1 is a list of some known fully functional glove box or full suit laboratories actively pursuing BSL-4 research. Increase in BSL-4 containment laboratories world-wide directly correlates with dramatic increase in biodefense funding from the beginning of the decade to present day. 

**Table 1:** Laboratories with Active biosafety level 4 (BSL-4) Research Programs.

Location (alphabetical by country)	Laboratory Name(s)
Geelong, Victoria, Australia	Australian Animal Health Laboratory, Commonwealth Science and Industrial Research Organization
Minsk, Belarus	Republican Research and Practical Center for Epidemiology and Microbiology
Winnipeg, Manitoba, Canada	National Microbiology Laboratory, Canadian Science Centre for Human and Animal Health
Lyon, France	Jean Mérieux BSL-4 Laboratory, French National Institute for Health and Medical Research
Gabon, Africa	International Center for Medical Research, Franceville
Marburg, Germany	Philipps University of Marburg
Hamburg, Germany	Bernhard-Nocht Institute for Tropical Medicine
Bhopal, India	High Security Animal Disease Laboratory (HSADL)
Pune, India	National Institute of Virology
Moscow, Russia	Center for Virology, Sergiyev Posad
Koltsovo, Russia	Russia’s National Research Center of Virology and Biotechnology in Koltsovo (VECTOR)
Johannesburg, South Africa	Special Pathogens Unit, National Institute for Communicable Diseases
Solna, Sweden	Swedish Institute for Communicable Disease Control
Porton Down, Wiltshire, United Kingdom (UK)	Health Protection Agency (HPA) and the Defence Science and Technology Laboratory (Dstl)
Colindale, UK	Health Protection Agency's Centre for Infections
London, UK	National Institute for Medical Research
Potters Bar, UK	National Institute for Biological Standards and Control
Atlanta, Georgia, USA	Centers for Disease Control and Prevention
Fort Detrick, Maryland, United States of America (USA)	United States Army Medical Research Institute of Infectious Diseases (USAMRIID); NIAID — Integrated Research Facility (NIAID IRF); National Biodefense Analysis and Countermeasures Center (NBACC)
Galveston, Texas, USA	University of Texas Medical Branch at Galveston (UTMB) Shope Laboratory and Galveston National Laboratory (GNL)
Hamilton, Montana, USA	NIAID Integrated Research Facility (IRF) Rocky Mountain Laboratories (RML)
San Antonio, Texas, USA	Texas BioMedical Research Institute (Texas BioMed)

One of the common goals of research at these facilities is to pursue licensure of approved medical countermeasures that protect against exposure to BSL-4 agents. In the United States, all testing and evaluations for licensure must adhere to Good Laboratory Practices (GLP, 21 CFR Part 58) guidelines and the US Food and Drug Administration’s (FDA) Animal Rule (21 CFR 314.600), which states all licensure and product development must be performed in a well-established animal model that closely mimics the human disease course for a particular agent including route of exposure [7,8]. The parameters outlined above are not easily accomplished without sustained collaborations between government, industry and academic research partners, as well as extensive planning and resource management of BSL-4 facilities. GLP studies require more personnel, extensive documentation and undivided attention to detail. GLP studies often compete with a facility’s ongoing non-GLP basic research for space, personnel and resources. BSL-4 laboratories are tightly controlled research environments, impose physical limitations on the research staff, consume large amounts of time and labor, and are expensive to maintain. Performance of GLP studies at BSL-4 is a complex undertaking, for all validation and qualification activities performed on assays and equipment must be completed, and subsequently maintained, at BSL-4 conditions for successful execution of GLP compliant studies. Despite these limitations, highly innovative scientific investigations utilizing modern methods and instrumentation are being used to develop vaccines and therapeutics against highly pathogenic agents in BSL-4 containment laboratories. This review will explore the impact of regulations, safety considerations, and physical limitations on research progress in BSL-4 facilities within the United States. 

## 2. Gaining Access to Work in BSL-4 Containment Laboratories

### 2.1. Personnel Approval for Access

Following the aftermath of September 11, 2001, government institutions and universities were under scrutiny to develop more defined measures to screen individuals for gaining access to biocontainment laboratories and BSAT. Individuals are currently screened for overall good health, typically with a thorough medical physical examination which may include chest x-ray, electrocardiogram (ECG), basic blood work, vision test, hearing screening, urinalysis for illicit drug and alcohol usage, and most recently, behavioral health screenings. Behavioral health screenings were designed to identify workers exhibiting behaviors that could potentially threaten a nation’s safety and to gauge their opinions on laboratory safety in high containment and personnel reliability [3,9]. In the United States, the DOD’s Personnel Reliability Program (PRP) is in place to continuously monitor individuals for changes in health, mental status, and drug and alcohol usage. Similar institution specific programs are in place at other institutions with biocontainment laboratories. Medical and behavioral health screenings can generally take 1 to 2 months or longer, depending on the institution and the health status of the individual. 

In addition to the health screenings, individuals seeking approval to work with BSAT are subjected to federal background investigations. These investigations can take anywhere from 3 to 24 months depending of type of clearance sought, previous or existing clearances, personal history and type of government institution or university requesting the clearance. Government institutions and universities vary on which investigation will be conducted, and time required for the process can vary. In the United States, the most comprehensive background investigation is the single scope background investigation, or SSBI. This investigation allows an individual to receive a top secret clearance and follows the Office of Personnel Management form SF86 Questionnaire for National Security Positions. Information investigated includes verification of an individual’s identity, previous and current residences; work history, education, references, foreign travel and contacts; psychological and emotional health; criminal and financial records; illegal use of information technology systems; illegal use of drugs and alcohol, and association with terrorist related organizations. In lieu of the SSBI, other institutions may only require a unique Department of Justice (DOJ) number and a security risk assessment (SRA). The institution’s responsible official (RO) submits an Animal and Plant Health Inspection Service (APHIS)/CDC application Form 1, section 4 to their lead agency, either APHIS or CDC requesting to add the individual to the institution’s existing registration. The lead agency issues a letter to the RO with a unique DOJ number associated to the employee seeking access to work with BSAT. The employee can then complete Form FD 961 (Bioterrorism Security Risk Assessment Form), obtain two sets of fingerprints and forward all of this information to the Federal Bureau of Investigation (FBI)’s Criminal Justice Information Services (CJIS) Division. Once the investigation is successfully completed, CJIS informs CDC division of select agents and toxins (DSAT) to issue a letter stating successful completion Security Risk Assessment and authorizing work with Select Agents and Toxins. An SRA is valid for 3 years. 

### 2.2. Personnel Training Process

Once the individual has completed and passed the screening processes, and received the appropriate clearance and authorizations, the laboratory worker may now request physical access to the laboratories with BSAT [10]. Physical access necessitates three elements of training, each provided at some level depending on the institution: theoretical training on the principles of biosafety, laboratory orientation/facility training, and one-on-one mentorship for BSL-4 hands-on training. Some institutions may require additional classroom-type training, including instruction on procedures employed in the BSL-4 laboratories and the basic properties of agents handled in the laboratory. Others require an additional 3 week BSL-4 course, which thoroughly reviews the Biosafety in Microbial and Biomedical Laboratories (BMBL) 5th Edition procedures for select agents and BSL-4 practices [1]. Additionally, the course addresses normal BSL-4 operations such as suit practices, user mobility, laboratory work, data entry, animal handling, and safety practices. 

Some government facilities and universities require prior biosafety level 3 (BSL-3) laboratory experience before allowing access to the BSL-4 containment laboratories. Helpful techniques to learn at BSL-3 include: animal manipulations such as blood sampling, administration of test articles and vaccine doses, health status observations, administration of anesthesia or euthanasia agents, oral gavage and gross necropsies. *In vitro* assays are reviewed with the trainee, such as cell culture-based drug screens, plaque assays, propagation of viral stocks, basic microbiology and virology techniques, and the use of specialized equipment such as ultracentrifuges and blood analysis machines. Institutional committees and the laboratory’s BSL-4 management generally review the worker’s experience to ensure adequate time spent and experience gained in BSL-3. If the committee and management believe the individual has spent enough time in BSL-3 and accomplished the tasks they wish to perform in BSL-4, they may be granted a BSL-4 mentorship. Before they enter the BSL-4 space, the worker will receive lab-specific training and positive-pressure suit training. Currently, institutions in the United States are using positive pressure suits, such as the Dover CHEMTURION encapsulating suit (manufactured by ILC Dover, Inc., Frederica, DE, USA) and the Sperian protection suit (manufactured by Honeywell/Sperian/Bacou Technologies, Lyon, France). During suit training, the worker will learn about the construction of the suit and the air filters associated with each one, how to properly change gloves, pressure checking of the suit to ensure there are not any leaks or tears present, and dexterity exercises. A mentor(s) will be chosen for who will directly supervise the trainee ensure he or she learns and can easily follow proper procedures for entering and exiting the BSL-4 suite, providing guidance for laboratory procedures, and providing safety oversight for any life‑threatening and non life-threatening emergencies that may arise. The trainee, accompanied by the mentor must complete many entry and exit cycles and log numerous hours of supervised training in BSL-4. The trainee should progress from observing specific techniques to assisting with techniques to finally performing the technique by him or herself under the direct supervision of the mentor. After the trainee has become proficient in the BSL-4 containment laboratory and has met institution-mandated milestones, the trainee presents his or her completed training record to the committee and laboratory director to review. If the committee and laboratory director agree the trainee is comfortable with all aspects of BSL-4 including checking the suit before entry, proper entry and exit procedures, emergency procedures, laboratory-specific techniques, and general laboratory upkeep, the trainee will receive written approval for independent access. If the new BSL-4 worker chooses to learn additional techniques in BSL-4 that he or she had not experienced during the mentorship process, they can choose an approved mentor that is willing to train them on the task. The mentor again supervises the new worker until they are comfortable performing the task on their own. As continuous training by peers occurs, the worker will maintain an ongoing record of any additional tasks learned and training received. As this new BSL-4 worker gains more time and experience working in the suite, he or she may ask to serve as a mentor and to train the next group of BSL-4 researchers.

## 3. Routine Entry Process from the Common Hallway to BSL-4 “Hot Side”

The process of physically entering the BSL-4 containment suite and coordinating the ingress of supplies takes time, and varies depending on the standard operating procedures at individual facilities. The following section generally describes the process in US laboratories. Some facilities pass laboratory supplies or animals in cages through the hallway-airlock entry to the grey side. Anesthetized nonhuman primates in transfer boxes may also enter through this airlock, a process which must be executed quickly due to the limited time during which the animals may remain anesthetized. Other facilities are equipped with air pressure resistant (APR) doors, which allows for the direct passage of animals into an approved animal room that is under BSL-2 conditions. The animals are then allowed to acclimate and the APR door is locked from the BSL-2 side. The inner APR door from the BSL-4 side is then released and the animals transition from BSL-2 to BSL-4 for the current study. After personnel entry through a security access point into the outer change room, or the “cold” side, from the common hallway, a researcher must remove all personal items of clothing, undergarments and jewelry, except for eyeglasses, if necessary. Facility-provided laboratory clothing, basically long-sleeved surgical scrubs and socks, are donned before entry into the transitional side or “grey” side via badge reader, personal identification number (PIN) code and sometimes a biometric reader such as a fingerprint or retinal scanner. The method of personnel access is dependent on laboratory design at individual facilities. Once on the grey side, the process of donning additional PPE, checking laboratory safety features and confirming suit integrity begins, and may follow an institute checklist. Required PPE typically may include: earplugs/hearing protection (e.g., for the Dover CHEMTURION suits), one or more pairs of latex or nitrile exam gloves (sometimes taped to scrub sleeve cuffs), optional taping of socks to scrub pant cuffs, and an optional hair covering. Daily suit preparation involves visual inspection and manipulation of the suit and the outer gloves for defects. The outer suit gloves may be hefty neoprene, nitrile or latex gloves, generally 15 to 30 mil (millionths of an inch) thicknesses, and appear similar to dishwashing or canning gloves. Outer suit gloves are changed depending on the procedures employed by the institution, which may be every three entries, or at least once per week or more often if necessary, by using wide waterproof tape to attach the glove at the cuff of the suit sleeve. When gloves are changed, a suit pressure test is performed which enables the user to confirm no air leakages or holes in the suit fabric, which most frequently appear at gloves or on suit feet. Suit checks may be recorded as entries in a suit maintenance logbook. Depending on the institution, any damage found or repairs made may be reported to an institute’s safety office for consideration of appropriate action or at minimum, databasing individual suit integrity. Once suit integrity is confirmed, the suit is donned and connected to an air hose on the grey side, before BSL-4 or “hot” side entry, to confirm functionality of the air line and the coupling mechanism on the suit. 

Procedures and features for entry and exit into the hot side may differ depending on the institution. When closed, the grey-side chemical decon shower door is held shut by an electromagnet and an inflated gasket surrounding the entire door (APR door), which stays inflated to block any air transfer. Access into the shower vestibule may require a PIN code, biometric reader or other possibly unmonitored opening mechanism, which deflates the air gasket bladder and disengages the magnet. The grey-side shower door is opened and the researcher, along with any supplies or animals, then enters the shower vestibule, closing the grey side door behind them. Once the grey side door magnet and air gasket are re-engaged, the BSL-4 or hot side shower door, also equipped with a magnet and air gasket, is opened. All supplies are unloaded into the hot side. Some institutions require the use of protective over-boots or foot coverings, which are stored on the hot side and donned on entry, and other places may even require the use of a third pair of gloves over the outer suit gloves. Once the shower vestibule is empty, it must be decontaminated after the hot side door is closed and before another researcher enters from the grey side. To do this, the decontamination shower is initiated, the hot side door is closed through full engagement of magnets and gaskets, and the empty shower completes its run cycle. The shower is then decontaminated for the next person who enters from the grey side.

After work is completed, exit procedures may follow the reverse order from the entry process, depending on the standard operating procedures at the institutions. In general, the hot side shower door is disengaged to allow entry into the shower vestibule. The over-boots, if used, are removed and scrubbed clean. The researcher closes him or herself in the chemical shower, connects to air, and runs the decontamination shower. The shower runs for multiple cycles, which includes at least a several minute cycle of disinfectant and a several minute cycle of water. During the shower cycle time, sponges and brushes are used to clean and scrub the outer suit surfaces, built-in boots on suits, and gloves. At the completion of the shower cycle, the researcher re-enters the grey side where the suit is doffed and hung to dry and PPE are disposed into a biohazard bag. Laboratory clothing is removed and placed into a laundry bag for autoclave sterilization and laundering once removed. The researcher then exits through the personal shower, before putting street clothes back on and exiting the cold side change room.

When the entire entry process is performed uninterrupted and only few supplies are taken in, it can be accomplished in as few as 15 minutes. Uninterrupted exit from the BSL-4 side to cold-side/hallway may take about 20 minutes. Some facilities have gender specific change rooms to avoid delays incurred by waiting for a colleague of the opposite sex. Changing gloves and pressure testing the suit adds another 15 to 30 minutes to the entry process, and waiting to coordinate with coworkers for entry or exit as a team can take longer yet. Depending on the level of activity and number of personnel entering or exiting the lab, a person might have to wait through a cycle of the decontamination shower before entry or exit to/from the hot side. 

## 4. Laboratory Environment and Work Flow at BSL-4

### 4.1. Time Considerations

In general, work progresses more slowly at BSL-4 compared to BSL-2. Biosafety is a priority for all biomedical research labs no matter what the level of biocontainment, and all work must proceed at a careful and deliberate pace, with limited multitasking and no rushing to complete tasks. The positive‑pressure suit must stay inflated at all times to be an effective safety control, therefore an investigator must move around the laboratory and perform work while connected to an air hose, and movement may require multiple dis-/re- connections to hoses. It is difficult to perform many finer tasks with much deftness in a bulky and cumbersome suit, boots, and gloves. The suit and boots add weight, which can be tiring after long periods of time. It should be noted that some institutions have personnel time limits at BSL-4 to prevent worker exhaustion and any ensuing mistakes which could lead to accidental exposure. Hands are clad with at least two layers of gloves, the inner latex/nitrile exam glove, plus the outer suit glove, which is always larger than the hand size for ease of donning and doffing, and usually has extra glove material extending past the ends of fingertips, even with optimal fit-size. The combined glove material makes performance of finer tasks, such as capping 500–1,000 tubes of individual 1 mL aliquots of virus stocks, handling tiny items, taping or sticker- labeling items and writing on tubes infinitely more difficult. Handling items such as sterile cell culture plates and flasks and anything that requires finesse is difficult due to bulky gloves and the wide cuffs on the suit sleeves. It is prudent to prepare everything possible on the BSL-2 side, and carry items in that are ready for use, such as pre-labeled tubes. 

For the setup of work, the workspace inside the class II biosafety cabinet must be equipped with kill pans filled with disinfectant solution, biohazard bags to receive waste, absorbent towels and bottled disinfectant for cleaning as work progresses. Frequent surface decontamination of gloves with disinfectant, and decontamination of virus culture vessels as soon as they become waste, is common work practice. Different institutions may have various protocols for handling and decontaminating infectious solid and liquid waste. All sharps, including pipette tips and serological pipettes, and especially needles, are disposed of carefully to prevent puncture of containers or PPE. Instruments that enable liquid handling, such as ELISA plate washers and 96-well or 384-well robotic dispensers can be very helpful, since carefully pipetting many small volumes into culture plates can be difficult work wearing thick gloves. Another timesaving apparatus is the attachment of a camera to an inverted microscope for visualization of cell cultures. The face shield of the suit makes it very difficult to see into the eyepieces of a microscope, because placement of both eyes in front of the eyepieces is hard to achieve behind the flat plastic of the face shield. Instead, a live or photographed image of the cell culture taken through the camera and viewed on a computer monitor can simplify a task of looking at and recording data in multiple wells on a 96-well plate. One drawback to timesaving instruments is the footprint of space they require, for BSL-4 laboratories generally have very little unoccupied counter or tabletop space, if any.

### 4.2. Restricted Supplies and Equipment

Certain items should be carefully considered before use in biocontainment. Fragile items or items made of glass should not be used inside BSL-4 labs, if possible. Frequently certain reagents, such as Trizol® (Life Technologies, Grand Island, NY, USA) or reactive chemicals are supplied in glass bottles which have to be completely coated with laboratory tape before carrying into the containment lab to avoid breakage. Glass test tubes and Pasteur pipettes are restricted, and glass slides should be handled with extreme care; these types of items should be replaced with plastic alternatives. Generally there is little need for glass beakers or specialized chemistry glassware, as there are limited numbers of experiments, if any, which require such specialized supplies. Certain pieces of equipment used for aerosol experimentation, such as an impinger or aerosol generator, are made of glass and are required for the procedure. These items cannot be wrapped or protected so the utmost care must be used when working with these exceptions. Special consideration should be given to pieces of equipment that could generate an unwanted aerosolization of virus fluids, such as a pipettor or a centrifuge. Generally, cuphorn sonicators and small portable vacuum pump devices fitted with HEPA filters may be used in limited capacities at some institutions. Small tabletop centrifuges, cell sorters, plate washers or any other liquid-handling equipment that may generate an aerosol should only be used when placed inside Class II biosafety cabinets during operation, if possible. Any equipment that generates heat, such as a hot stir plate, a heat block, a heat sealer or Bunsen burner must be operated with extreme caution and safety awareness to avoid burns of the gloves or suit or other laboratory fire hazards. 

### 4.3. Virus Sample Fixation and Inactivation

Removal of any infectious or potentially infectious stock virus suspensions, biological samples, tissues or cell-culture supernatants from biocontainment, for use as a reagent in further studies, requires inactivation by heat inactivation, radiation or chemical means. For inactivation methods such as gamma irradiation, kill curves should be established for each virus to identify the amount of radiation required for inactivation. Furthermore, the inactivation method must be proven to be effective through safety testing to confirm absence of any remaining live virus in the sample. Once a given inactivation method is known to work, then it can become part of the institute’s standard operating procedure, and safety testing may not be required on every new sample inactivated in this manner. For example, dissected tissues (1 cm^3^) harvested from an infected animal at necropsy can be placed in 10% neutral buffered formalin and left in to inactivate for no less than 21 days in most facilities, although methods requiring alternative fixatives and shorter timeframes have been adopted at some institutions. During the fixation timeframe, multiple fixative exchanges may be required before removal from BSL‑4. At the end of the time period, it is accepted that these tissue samples are inactivated, and they can be removed from containment for further histological processing without sampling them directly for live virus by a safety test. 

A safety test for live virus consists of designing an experiment with all proper positive and negative controls to test for the inactivation potential of a reagent (such as a denaturing buffer in a kit), a novel fixative that might be alcohol-based instead of formalin based, or a proposed disinfectant [11]. Depending on the inactivating agent used, it may be necessary to include a dialysis step prior to safety testing to remove residual inactivating agent, and this process can add time and effort to overall safety testing processes. The following procedure is an example of a thorough safety test. Samples of virus fluids or solids inactivated with the new reagent under test are allowed to incubate to completion and then subjected to two or more passages, or amplification cycles, on a cell-culture monolayer permissive for virus replication, in an effort to replicate any remaining virus in the sample. Once the amplification test has had a chance to grow for 4 to 7 or more days, depending on the pathogen, it is passaged onto a second monolayer for 4 to 7 or more days, and then the supernatant from that amplification must be tested by appropriate virus detection methods, such as plaque assay, immunofluorescence test or RT‑PCR to look for the presence of live virus, virus antigen, and/or virus genomic material. Absence of virus material, compared to well-designed controls, indicates that the inactivation was successful and this new inactivation method can be approved by the institute’s Safety Office, and adopted for use in future experiments. Safety tests can take up to 1 month to complete, depending on the virus, and must be performed in triplicate to ensure inactivation, so development of these methods can be time consuming. A variety of inactivation methods are routinely in use. Table 2 demonstrates some inactivation methods, the chemical nature of the reagent, and the time required for inactivation.

**Table 2:** Some commonly used chemical agents and methods of virus inactivation and decontamination.

Inactivating agent	Chemical nature of inactivating agent	Method	Time to sample inactivation and removal from suite
Trizol® Reagents (Life Technologies, Grand Island, NY, USA), or similar [11]	Phenol, guanidinium isothiocyanate	Mix one part sample to 3 parts Trizol®. Treat the threads of the vial with Trizol® before sealing. tube.	Immediate
Qiagen RNA lysis buffers [11,12]	Guanidinium chloride plus ethanol or phenol	Mix one part sample to at least 3 parts lysis buffer. Treat the threads of the vial with buffer before sealing.	Immediate
Gamma irradiation [13]	CO_60_ radiation	Placement of sample in irradiator and exposure to a known number of rads (e.g., 1 million or more)	Radiation treatment plus confirmatory safety testing on a sample must be completed
Heat, plus SDS sample buffer [14]	Heat and denaturation by detergent	Boil sample for 5 minutes in 2%–4% SDS sample buffer	Immediate
10% neutral buffered formalin (NBF) [15]	Formaldehyde fixative	Submerge tissues, cell culture plates or samples on slides in formalin. Must be a 10:1 formalin to sample volume ratio, and any jar threads must be wiped with formalin upon sealing lids. Multiple exchanges of formalin buffer must occur for necropsy tissues.	Necropsy tissues must inactivate for 21 days. Cells on plastic culture dishes or slides must inactivate no less than 24 hours
0.1%–1% glutaraldehyde, 4% paraformaldehyde with/out 1% osmium tetraoxide [16]	Fixatives common to microscopy techniques	Immersion of sample in fluid, or exposure to vapors	At least 24 hours
Fresh 1:10 dilution of bleach	5.25% sodium hypochlorite	Disinfectant for surfaces	Not a tissue or sample preparation reagent
Ethanol	70% solution of ethanol	Disinfectant for surfaces	Not a tissue or sample preparation reagent
5% Micro-Chem Plus^TM^ (National Chemical Labs, Philadelphia, PA, USA) in water	Proprietary blend of dimethyl ammonium chlorides and polyethylene ether glycols	Disinfectant for surfaces, showers	Not a tissue or sample preparation reagent

Inactivated samples, such as tissues in formalin or blood samples in tubes of Trizol® can be removed through the decontaminating chemical shower or through a disinfectant chemical dunk tank (if available). Tissues in plastic formalin jars, or tubes containing inactivated material must be well labeled, for the exterior of tubes must be subjected to a disinfectant bath (5% Micro-Chem Plus, or equivalent) for removal from the suite. Cell culture plates inactivated for at least 24 h in 10% formalin can be removed after packaging in heat-seal bags containing fresh formalin to bathe the plates. Only a few items should be showered out at a time, due to the time it takes to wash each item in the decontaminating chemical shower. Inactivated samples removed from containment may need to be recorded as to type, quantity and storage location, for inventory purposes.

### 4.4. Entry and Removal of Equipment

Equipment can be taken in and removed from the BSL-4 lab through a sealed airlock chamber where timed-release, vapor-phase decontamination can be set up in advance. Generally, equipment used on the BSL-4 side which may need to be removed for maintenance or repair will be disconnected and any tubing or chambers treated with disinfectant solution as appropriate. The instrument is then wheeled into the airlock vestibule through a door which is sealed using similar air-gasket and magnet technology to that described for the decontamination shower for personnel entry and exit. A formaldehyde gas, chlorine dioxide or hydrogen peroxide vapor treatment for a requisite period of time is generally used to decontaminate the surfaces and insides of equipment [1]. Successful decontamination of equipment, or entire laboratory area for times of shut-down, must be demonstrated by a verification assay, such as a spore inactivation test. Once the vapor treatment is complete, and the levels of gases are safe for personnel, the BSL-2 or hallway-side door of the airlock can be opened for equipment removal. New or repaired equipment is taken into the BSL-4 lab following the same process in the reverse order. Once inside the BSL-4 side, the vestibule is re-sealed, and the empty airlock is decontaminated using the chosen vapor method. 

### 4.5. Communication and Data Handling

Communication with coworkers both inside and outside of the suites is vital to successful performance of laboratory work. Importantly, telephone, visual information marquees, visible and audible alarms, fax machines and computers are generally available to assist with daily communication as well as emergency communication. Verbal communication is difficult because the ILC Dover CHEMTURION blue suits are noisy (up to 85 decibels) when connected to an air hose, and therefore the wearer is also equipped with earplugs. When wearing a blue suit, it is easiest to communicate in person or by telephone only when disconnected from air, writing messages or as a last resort, pantomiming information. Some laboratories have suits equipped with microphones and radios for easier communication. Windows between the laboratories and common building hallways allow for conversations via written messages, visual demonstration of results or sometimes animal health assessments, depending on placement of animal cages with respect to the windows. BSL-4 labs may be equipped with security cameras that can serve the purpose of observing workers from a control center, maintaining lab security, observing any potential emergency situations, or even sometimes serving as a mechanism by which work can be audited by an institution’s Quality Assurance Unit.

Access by workers in containment to networked computers is essential for email communication and data circulation. Computers attached to an instrument such as a plate reader or flow cytometer must be networked to allow the data to be accessed outside of the containment suite. If networking is not available for a particular instrument due to its technology, then data can be recorded on a compact disc, or printed on paper. Flatbed scanners can be used to generate electronic document files of any data recorded on regular paper, and for the purposes of GLP research, these electronic files can be certified as true copies and the original paper data can be destroyed when no longer needed. Some BSL-4 labs have used a computerized data capturing system in animal studies, which allows for faster and easier data collection and handling [17]. Further, some of these data collection systems are GLP compliant. Space in BSL-4 labs for storage of paper records is essentially non-existent, and these original pages could never leave the laboratory for direct archiving. Plastic paper, upon which data can be printed or written with indelible ink is available for purchase (PolyPaper, Nalgene, Rochester, NY, USA), but it is expensive, and the quality of the data recorded on the paper may not be long‑lasting and therefore unsuitable for archival records. Plastic paper, if used, is excellent for small scale note-taking, general research use, and serving to keep the researcher organized. This paper can be removed through the decontamination shower at the end of the work period, and maintained as research notes in a laboratory notebook outside of containment. It is only possible to decontaminate a limited number of sheets of plastic paper per decontamination shower, due to shuffling and handling in a shower environment and ensuring that each sheet gets properly washed during the shower cycle. If necessary, compact discs of data can easily be surface decontaminated through the chemical shower and read outside of containment. Refrigerator and freezer storage space can be limited, especially for longer term storage of biological samples or GLP samples, when necessary. Plans for proper storage can impact availability of laboratory space and costs of setting up facilities, and should be considered at the outset of laboratory design.

## 5. Execution of Animal Studies in Biocontainment

### 5.1. Animal Studies

As mentioned above, animal studies are used to evaluate the efficacy of candidate vaccines and therapeutics against highly pathogenic viruses handled at BSL-4 containment. Animal studies at BSL-4 carry more risk, but the animal handling methods are similar to those followed at BSL-2. Animal caretakers, technical support staff and pathologists undergo extra training to work safely with infected animals and contaminated animal samples and waste products. Small animals, such as mice, hamsters, and guinea pigs, may be housed in microisolator cages with filter top lids. Challenge of animals with BSAT, or daily dose administrations to infected animals requires training and utmost care to avoid accidents [18]. Slow and careful work is the best way to avoid a needlestick exposure during animal challenge or treatment work. Animals are cared for daily by a dedicated animal caretaker, and researchers with responsibility for the animal’s health and wellbeing must perform health checks once, twice, or more often per day, depending on the needs of the animals on study. Humane euthanasia is provided for any animal exhibiting signs of disease from which recovery is unlikely. 

Nonhuman primate (NHP) studies follow these same general principles. Monkeys are acquired and sometimes implanted with telemetry devices or catheters, depending on experimental needs. Gender and weight data are provided to a statistician for randomized assignment of individual animals to study groups. In a process that generally takes half of a work day, anesthetized monkeys are transported into the BSL-4 suite generally one week before the study begins, to ensure acclimation to the new environment and reduction of animal stress, which could adversely affect the study results. At BSL-4 NHPs are generally housed in individual cages equipped with squeeze mechanisms to immobilize the animal when necessary for observation or administration of challenge or test article. NHP are anesthetized for all manipulations outside of the cage including delivery of virus inoculums by aerosol or parenteral exposure, blood draws, and intravenous administration of test articles. Blood sampling and processing for hematology, clinical chemistry and coagulation parameters for a 24 to 30 monkey study consumes an entire workday, employing a team of four to six technicians of various skill sets depending on the number of samples to process and store (typically at −80 °C). The in-life phase of a study may last for up to 45 days, since recent guidance from the FDA has indicated that the agency would like to see animals observed for four–five times the length of the disease period after recovery, to ensure no disease recrudescence. As a consequence, NHP studies supporting advanced development studies under the FDA Animal Rule can be longer, more costly, and take more space resources in BSL‑4 laboratories. After the in-life phase of a study has been completed, plaque assays, ELISAs for antiviral antibody responses and any other biomarker assays may be performed on banked serum and tissue homogenates. On a complete sample set from 24 to 30 NHP, 3 to 4 solid days of ELISA assays and another 5 to 6 days can be spent performing the technical work associated with viral plaque assays and biomarker quantitation assays. 

### 5.2. Pathology and Necropsy Support

Another capability of extreme importance to support *in vivo* research at BSL-4 containment is the ability of trained pathologists to perform complete necropsies. Collection of animal study data revealing gross or histopathological changes is required, especially for NHPs, which are on research studies or GLP protocols in support of Animal Rule studies. Tissues from animals which succumbed to viral infection are heavily contaminated and must be handled carefully to avoid accidental exposure of the pathologist during necropsy procedures. Pathologists and other trained support staff complete extra training to safely perform necropsies at BSL-4, where they have to wield sharp or heavy instruments, such as scalpels and bone saws, to cut into highly infectious materials. There are challenges beyond just safely cutting and manipulating such infectious tissue. Frequently a large set of control animals may all require euthanasia on the same day of the study, creating a large number of necropsies that must be performed in a timely manner by a limited number of experts capable of completing the work. The work must be completed before the tissues begin to autolyze and decent tissue samples can no longer be obtained for histopathological analysis. Necropsy of each animal takes an hour or more, tiresome work if two to three sequential necropsies must be performed. For safety purposes, pathologists may be limited to three necropsies in a session on any given day, to avoid accidents which could occur due to hand fatigue and continual hours of work. As a safety precaution, cut‑resistant metal mesh gloves are generally worn over the outer suit gloves, and underneath some latex exam gloves while performing necropsies, and working wearing this additional PPE is tiresome to hands and arms. A necropsy assistant is generally required to weigh tissues, take notes, record data and assist with placing specimens into fixative. Because of these personnel limitations, significant consideration must go into study design for ultimate collection of robust and useful histopathology data.

## 6. Current Capabilities at BSL-4, and Newer Technologies

Some types of experiments do not need to be performed under BSL-4 conditions; therefore, capabilities for performing these methods are not usually maintained at high containment. Mass spectrometry and HPLC is not usually required at BSL-4, since sample treatment methods to isolate compounds and proteins are stringent enough to inactivate virus. Molecular biology methods such as DNA or RNA isolation, amplification, cloning, electrophoresis and sequencing all involve genetic materials which can be isolated through phenol/chloroform extraction methods and brought out of the BSL-4 in Trizol® or a similar reagent. Protein analysis experiments such as Western blotting, polyacrylamide gel electrophoresis (SDS-PAGE) analysis, and immunoprecipitations do not need to be carried out in high containment, for denaturing sample buffers and treatments will inactivate virus or cell proteins for removal and further manipulation. However, non-denaturing or native PAGE methods should be performed under BSL-4 containment conditions. Basic cell culture and plating of uninfected cells for use in plaque assays or virus culture assays can be completed in typical BSL-2 cell culture labs, and culture plates can be carried into the BSL-4 laboratories at the time of use. Preparation of tissue specimens in formalin or glutaraldehyde for further analysis by histology or microscopy methods must occur within the suite, but once the cells are fixed, all tissue embedding, sectioning, and histology, immunohistochemistry, electron microscopy methods occur in specialized pathology or microscopy laboratories, rather than inside the BSL-4 containment laboratory.

Experiments performed with live virus must be performed in containment. In addition, experiments to assay samples which came from infected animals or those animals which have recovered from infection must also be performed in containment. Only samples which have been treated with proven methods to inactivate virus, or which have been safety tested for viral inactivation can be removed from BSL-4. Table 3 below illustrates types of experiments or activities which must be performed at BSL-4, and what alterations would need to be made to enable the work to be done outside the suite, if possible.

**Table 3:** Methods involving live virus work have very few alternatives for performance at Biosafety level 2 (BSL-2).

Method or Assay	Alternative
Live virus quantitation: plaque assay, TCID_50_, focus forming or live cell assay for viral titration of a virus stock or an experimental sample	Any live virus quantification in samples must be done inside the suite. Plaque assays could be counted on photographs rather than inside the suite, but the assay must be performed entirely at BSL-4. Viral genomic burden in a sample can be measured by qRT-PCR.
Neutralization assay for antibody or chemical activity against virus	Plaque-reduction neutralization tests on native viruses must be done inside the suite. Use of BSL-2 pseudotyped or surrogate viruses, if available, could be substituted for testing antibody binding.
ELISA or plate-based assays using any reagent that is either live virus as substrate or capture antigen, or where the reagent (such as immune serum) under test could be potentially infectious	No immune serum can come out of the suite unless irradiated and safety tested. Irradiation may destroy serum proteins of interest before they can be assayed.
Flow cytometry on infected cells	Infected cells can be permeabilized and fixed in formalin and brought out for flow methods, provided that the antibody staining is not inhibited by the fixation.
Protein analysis methods	Some co-immunoprecipitation and native PAGE methods may require BSL-4 containment.
Cell culture based drug screening assays for antiviral activity	No alternative unless a pseudotyped or surrogate virus exists for use in screens.
Animal observations and veterinary assessments	No alternative, animals must be tended in person at BSL-4.
Quality Assurance audits supporting regulated (GLP) studies	No realistic alternative, most work cannot be closely audited from windows or security cameras.

BSL-4 laboratories at government or academic institutions have sophisticated equipment for use in *in vitro* or *in vivo* containment experiments (See Table 4). Many of these laboratories are equipped with equipment to aid in performance of hematological, clinical chemistry and blood coagulation analysis for preclinical studies. A few BSL-4 institutions have incorporated advanced medical imaging within high containment as part of their efforts to expand diagnostics, improve infectious disease research, and further develop medical countermeasures. Various imaging modalities exist depending on the institution’s capabilities and the parameters of interest. Ultrasound is a method of imaging that does not use radiation, rather; high-frequency sound waves which produce real-time, two or three‑dimensional images that allow for visualization of structures, organs, and tissues for diagnosis and analysis of abnormalities. X-ray images the body producing two-dimensional scans of electron dense tissues. X-ray fluoroscopy produces dynamic and static images of body’s anatomy. The images can be produced in real-time but contain limitations. X-ray fluoroscopy is utilized with Computerized Tomography (CT) to improve resolution, contrast and boundaries although using CT and real-time acquisition can be limited. Magnetic Resonance Imaging (MRI) utilizes a technique that does not use radiation and is noninvasive by incorporating magnets and radiofrequency pulses, which can detect chemical or functional activity, to produce three-dimensional images for identification and analysis of internal structures and functions of the body. MRIs can produce valuable information pertaining to anatomical, physiological, and biochemical changes within the body. Positron Emission Tomography (PET) makes use of a radionuclide labeled, metabolic probe which produces a three-dimensional image of the metabolic activity within the body. This method is often combined with CT, a method of three-dimensional anatomical imaging, to determine where the metabolic changes are occurring. PET allows for earlier detection of abnormal metabolic processes prior to that of abnormal anatomical changes. Single Photon Emission Tomography (SPECT) is another method that uses different radionuclide labeled, metabolic probes to produce a three-dimensional image of metabolic activity within the body. SPECT is combined with CT producing metabolic and anatomical activity for analysis. SPECT and PET vary on metabolic process desired, length of radionuclide decay, and image quality. Imaging provides an additional strategy for the study and evaluation of infectious diseases. 

**Table 4:** A wide variety of equipment exists at various BSL-4 laboratories for performance of sophisticated methods.

Type of equipment	Example of equipment used
General Laboratory equipment	Class II and III biosafety cabinets Temperature-controlled, humidified CO_2_ cell culture incubators of all types Refrigerators (2–8 °C), Freezers (−20 to −80 °C), Liquid Nitrogen storage Ultra-, super speed, table top and microfuge centrifuges Plate readers of various types, standard or specialized Plate washers Microwave Tissue grinders Water baths, if approved for use Chemical balances Autoclaves
Specialized immunoassay equipment	Sector® Imager 6000 (Meso Scale Discovery) Virocyt 2100 Virus Counter (Applikon Biotechnology) Flow cytometers, e.g., Fortessa (BD Biosciences) ELISPOT readers and bead/cell sorters
Animal treatment or handling equipment	Telemetry, e.g., DSI JET^TM^ (Data Sciences International) Aerosol exposure systems, e.g., AeroMP (Biaera Technologies) Animal weigh scales (large and small)
Clinical chemistry and pathology instruments	Serum chemistry machines such as Vitros® (Ortho Clinical Dignostics) Hematology machines, such as Advia® 120 (Siemens Healthcare Diagnostics) Coagulation machines, e.g., Sysmex CA-560 (Sysmex) Histology scanners Tissue digesters
Imaging instruments	Light boxes/digital camera setups Microscopes, such as inverted, fluorescence, or dissecting scopes, Luminex 100/200^TM^ (Luminex Corp.) Plethysmography equipment (Buxco Research Systems) Imaging equipment, such as MRI, PET/CT, Ultrasound, X-ray fluoroscopy, SPECT/CT High content imaging, e.g., (PerkinElmer)

## 7. Conclusions

This review summarizes and documents some of the biosecurity regulations, safety considerations, research space limitations, and physical limitations for personnel in the existing BSL-4 laboratories within the United States. While it may be a slow, difficult process to gain access and training to work independently at BSL-4, a researcher can perform a wide variety of interesting and crucial studies in this environment to aid in medical countermeasure development. The BSL-4 environment has evolved tremendously, especially in the last 10 years. Policies and regulations, though always present and important, have been elevated to higher stringency since September 11, 2001. Training, safety and security procedures are scrutinized and now well documented with various outside agencies performing scheduled or random facility and program inspections. The researchers in this environment have a responsibility to the general public as well as to each other to ensure the level of protection and quality of the research remains high. The limitations and hurdles to become a proficient BSL-4 researcher have been outlined. Simple tasks that can be accomplished within BSL-2 or -3 environments become much more cumbersome and require extensive planning, communication, time management and reliability when performed at BSL-4. Most tasks performed within BSL-4 have some type of standard operating procedure specific to the environment. Several BSL-4 institutions have been referenced in this review and have provided valuable insight into their high containment programs. Economic limitations and the scarcity of funding, require these institutions to work together and collaborate to minimize duplication of efforts as well as maximize knowledge, capabilities and equipment usage. Industry has also increased its partnership with governmental or academic institutions within the field of high biocontainment, towards development of useful products or advancing therapeutics and vaccines. Scientific vendors are more willingly working with the research staff to incorporate the state of the art technology within the BSL-4 environment to help alleviate the stresses and rigors of this type of research. Increasing awareness of proficiencies and deficiencies at BSL-4 can lead to more effective collaborations within the scientific community, with a goal of stronger discovery and therapeutics development research. 
